# Capturing doping attitudes by self-report declarations and implicit assessment: A methodology study

**DOI:** 10.1186/1747-597X-3-9

**Published:** 2008-04-21

**Authors:** Andrea Petróczi, Eugene V Aidman, Tamás Nepusz

**Affiliations:** 1 Kingston University, Faculty of Science, School of Life Sciences, Penrhyn Road, Kingston upon Thames, Surrey, KT1 2EE, UK; 2 The University of Sheffield, Department of Psychology, Western Bank, Sheffield, S10 2TN, UK; 3 University of Adelaide, School of Psychology, North Terrace Campus, Level 4, Hughes Building, SA 5005, Australia; 4 Research Institute for Particle and Nuclear Physics of the Hungarian Academy of Sciences, Computational Neuroscience Group, Budapest, 1121, Konkoly Thege Miklós út 29-33, Hungary

## Abstract

**Background:**

Understanding athletes' attitudes and behavioural intentions towards performance enhancement is critical to informing anti-doping intervention strategies. Capturing the complexity of these attitudes beyond verbal declarations requires indirect methods. This pilot study was aimed at developing and validating a method to assess implicit doping attitudes using an Implicit Associations Test (IAT) approach.

**Methods:**

The conventional IAT evaluation task (categorising 'good' and 'bad' words) was combined with a novel 'doping' versus 'nutrition supplements' category pair to create a performance-enhancement related IAT protocol (PE-IAT). The difference between average response times to 'good-doping' and 'bad-doping' combinations represents an estimate of implicit attitude towards doping in relation to nutritional supplements. 111 sports and exercise science undergraduates completed the PE-IAT, the Performance Enhancement Attitude Scale (PEAS) and answered questions regarding their beliefs about doping.

**Results:**

Longer response times were observed in the mixed category discrimination trials where categories 'good' and 'doping' shared the same response key (compared to 'bad-doping' combination on the same key) indicating a less favourable evaluation of doping substances. The PE-IAT measure did not correlate significantly with the declared doping attitudes (*r *= .181, *p *= .142), indicating a predictable partial dissociation. Action-oriented self-report expressed stronger associations with PE-IAT: participants who declared they would consider using doping showed significantly less implicit negativity towards banned substances (*U *= 109.00, *p *= .047). Similarly, those who reported more lenient explicit attitudes towards doping or expressly supported legalizing it, showed less implicit negativity towards doping in the sample, although neither observed differences reached statistical significance (*t *= 1.300, *p *= .198, and *U *= 231.00, *p *= .319, respectively). Known-group validation strategy yielded mixed results: while competitive sport participants scored significantly lower than non-competitive ones on the PEAS (*t *= -2.71, *p *= .008), the two groups did not differ on PE-IAT (*t *= -.093, *p *= .926).

**Conclusion:**

The results suggest a potential of the PE-IAT method to capture undeclared attitudes to doping and predict behaviour, which can support targeted anti-doping intervention and related research. The initial evidence of validity is promising but also indicates a need for improvement to the protocol and stimulus material.

## Background

The potential impact of sport to promote healthy lifestyle is seriously undermined by the presence of doping practices, which has spread beyond the elite sport [1,2]. The elevation of the use of performance enhancing drugs among adolescents [3-5] and even pre-adolescents [6] is a particularly worrying trend. To date, intervention strategies have appealed to moral values and health consciousness of the athletes. Effectiveness has typically been evidenced by changes in self-reported behaviour and/or explicit attitudes towards using prohibited substances. Despite the weak evidence, this approach is based on the assumption that athletes' actions are exclusively motivated by conscious cognitive processes. This may not be true. The fundamental questions to be addressed for effective intervention programs are: what is it that really drives highly skilled and motivated athletes to risk their health, reputation and future participation by engaging in doping practices? Why risk losing a future in one's chosen sport by committing an act that goes against the fabric of fair play and ethical behaviour? Are reasons always objectively evaluated and clearly articulated factors that can be measured by some explicit tools (e.g. questionnaires, interviews) or rather, may decisions also be influenced by values below the explicit awareness?

A whole range of factors impact on athletes' decision to take substances that are intended to provide a performance advantage. Some of these factors are well articulated by athletes themselves. The plethora of reasons athletes put forward to justify their doping practices is well known to the practitioners of sport psychology, whose case studies (e.g. Terry [7]) report reasons such as i) 'Everybody's doing it', ii) 'I don't want to but it's the only way to compete', iii) 'The doctors can't fix my injury, what other option do I have?', iv) 'I know what I'm doing, I won't get caught'; v) 'I still have to do the work' and vi) 'I'll do whatever it takes to win'.

Psychological analysis of doping behaviour has so far concentrated on individual differences in attitudes towards drug use [8,9] and towards drug testing programs [10]. What is not well understood are the underlying psychological mechanisms of the use of performance enhancing substance and methods in sport. Few studies have examined social and moral concerns [11] and achievement orientation [12] as potential candidates for such mechanisms. More recent reviews have catalogued co-morbidity factors such as ego-oriented achievement striving and motivational climate [13]; as well as narcissism, depression, lack of self-confidence, eating disorders, body image imbalance, dispositional propensity to risk-taking and suicide [14]. The role of attitudes in shaping behaviours has been widely recognised [15-17] hence understanding athletes' attitudes and behavioural intentions towards performance enhancement is critical to informing anti-doping intervention strategies.

Whilst athletes' attitudes and beliefs are central to most recent social science research into doping [4,18,19], they are typically measured by self-reports [20]. Capturing the complexity of these attitudes beyond verbal declarations requires complementary assessments using alternative methods. This paper examines the utility of one such method – the Implicit Associations Test (IAT) [21] – in assessing implicit doping attitudes in comparison to the self-report derived assessments. Its ability to capture deeply-rooted, more stable, unconscious or introspectively inaccessible representations could complement the traditionally used explicit assessments and make vital contribution to the understanding of drives behind doping behaviour.

The concept of implicit attitudes has been widely used in social psychology with a variety of measurement techniques [22] and its fundamental propositions have been supported with varied success [23]. Applications of implicit attitude measurement have increased in several areas, including health and exercise, where typical attitude targets include obesity [24-27], body weight in general [28], exercise [29] or health-compromising behaviours such as smoking [30], drinking [31,32] or drug use [33]. Implicit associations were contributed to the prediction of their respective behaviour in health compromising behaviour studies. Interestingly, while people with obesity showed a more positive implicit attitude towards food [29], alcohol studies evidenced the opposite: in general, heavy drinkers exhibit negative, neutral or ambivalent attitude towards alcohol in comparison to soft drinks but score higher on the alcohol-arousal [34-36].

However, when personalised implicit assessment was used, the implicit association with alcohol was positive suggesting that alcohol implicit assessment reflect negative extrapersonal knowledge [37]. It was evidenced in all cases that implicit association play an influential role in alcohol use and misuse.

Weak correlation/dissociation is expected for controversial attitude constructs, especially those associated with social stigma [38]. In the IAT literature, there are two fundamental explanations for the hypothesized weak relationship between implicit and explicit attitudes: i) response distortion (both intended and unintended, affecting explicit attitudes) and ii) the existence of dual attitudes. It has been assumed that implicit attitudes may be influenced by: i) early affective experiences, ii) systemic cultural view of the target, iii) cognitive consistency principles [39], iv) translation between the implicit-explicit representation, v) social desirability, vi) situational pliability and vii) research design issues [40]. This is consistent with the model of dual attitudes [41], which argues that a new attitude (acquired from experience later in life such as being in sporting situation where doping is present and manifest in explicit attitude) does not necessarily replace the older one but results in dual attitudes by holding different evaluations (implicit and explicit) of the same attitude object. It is assumed that the degree of co-influence of the implicit and explicit attitudes depends on the situation, the cognitive capacity to retrieve explicit evaluation [42] and many other factors.

On the contrary, the Iterative Reprocessing Model (IRM, [43]) provides evidence from fMRI investigations showing that while implicit and explicit attitudes serve different purposes, both automatic evaluations (implicit attitudes) and conscious self-reflection (explicit attitude) are not separated and both are equally important. The IRM suggests a continuous evaluative cycle between automatic evaluation (which occurs early in the evaluative process) and the relatively stable reflective evaluation (attitude). Assuming that both implicit and explicit association play an influential role in evaluating doping related situations, intervention strategies should target implicit an explicit doping related expectations.

The distinction between explicit and implicit attitudes raises the question of predictive power of implicit attitudes. This distinction is known to be highly task and context-dependent (for review, see Fazio & Olson [44]). Both explicit and implicit attitudes are involved in the decisions about behavioural intention and behaviour execution to varying degrees. According to Fazio's Motivation and Opportunity as Determinants (MODE) model [45,46], explicit attitudes are the amalgamation of implicit attitudes (automatic responses) and verbal responses to the attitude object affected by the motivation and opportunity to deliberate and respond in a strategic way.

The importance of implicit values in behaviour or behavioural intention is a key research question and attempts have been made to incorporate implicit attitudes into the traditional behaviour models [47,48]. Despite the fact that implicit attitudes are known to have stronger effects on behaviours that are less controlled by deliberate conscious processing [49], such as spontaneous responses, intuitive action or increased action readiness and sensitivity to the attitude-relevant situational cues, results obtained via implicit assessments, in combination with explicit measures, have improved the prediction of behavioural models in various domains requires deliberation [50-55].

At the time of writing, implicit attitude measurement has not been applied to doping attitudes. The study reported in this paper examined the validity of this doping-related IAT protocol (PE-IAT) by, first, ascertaining the magnitude of the anti-doping evaluative bias among sport and exercise science graduates and, second, by triangulating it against alternative estimates of doping attitudes. One advantage of the method is that it is not susceptible to deliberate response distortions (e.g. faking), which is especially important in doping related research [56-58]. Subjects trying to deliberately manipulate IAT do so by slowing their responses to desirable combinations but the improved IAT scoring procedures tend to cope with this strategy by eliminating very slow responses from the analysis. As the alcohol-related cognition studies [34-37] have shown, the IAT-type tests, however, can be contaminated by associations that are stored in memory but irrelevant to the individual's personal virtues [59,60]. Implicit assessments are thought to have the potential to tap into unconscious or introspectively inaccessible attitudes that are deeply-rooted in long-term socialisation [23].

### Assessment of doping attitudes

Despite their widespread use in sport psychology, self-report techniques have a range of substantial limitations. Most of these limitations stem from the following two assumptions: the test taker is assumed to (a) *be able *to self-report and (b) *be willing *to self-disclose. In other words, the test taker is assumed to have sufficient insight into what's being measured yet no intention to distort his or her responses. Accepting the assumption of the ability to self-report is a relatively safe bet, at least in dispositional trait assessment, as most trait markers tend to be universally understood [22]. The second assumption, however, remains wide open and as such, it should be scrutinised for each individual measurements. In particular, social desirability is known to contaminate questionnaire-based attitude measures, and this contamination tends to escalate with the increasing sensitivity of question content. Doping is a highly sensitive issue for those who are involved in sport, especially for those who derive their livelihood from it. Multiple pressures are likely to prompt athletes to conceal their attitudes towards doping if they are lenient. Not surprisingly, self-reported doping attitudes have shown a significant association with socially desirable responding, even when anonymous questionnaires are used [61]. Violations of either of these two assumptions can compromise the validity of self-report assessment and call for caution about relying solely on data derived from self-declarations [22].

### Towards an alternative attitude measure: Implicit attitudes and associations

Ideally, self-reporting methods should be complemented with alternative measurement techniques. One of the promising alternatives is the Implicit Associations Test (IAT) [21]. Based on the widespread definition of attitude as a relatively stable tendency to evaluate objects with degrees of liking or disliking, implicit attitudes are defined as associative processes reflecting this tendency [62]. Associative evaluations are the result of stimulus-driven, uncontrolled, unintentional, goal-independent or unconscious processes [63] and as such, they do not require respondents to be aware of these attitudes and thus can provide a solution for the problem of self-presentation distortion.

The IAT is capable of measuring automatic effects of implicit attitudes without relying on self-reports by extending the cognitive task-switching paradigm to the realm of timed semantic classification, typically in a semantical decision task. The use of semantic targets (words) opens the prospect of measuring the evaluative strength of a wide range of implicit associations between social objects [21]. The IAT assesses the generic differences in implicit attitudes by measuring the underlying automatic activation of cognitive processes. Initial validation of the IAT has shown its sensitivity to individual differences in implicit effects of self-esteem and self-identity [64], attitudes [21], and stereotyping [65-68], with no evidence of procedural limitations [22] or familiarity of stimulus [68] acting as confounding variables. The core of the method is a semantic discrimination task performed under time pressure: participants have to classify sematic targets (usually words or pictures) on computer screen into one of two opposing categories (e.g. pleasant vs. unpleasant) by pressing the respective response keys on the keyboard (e.g. right hand side – left hand side). The attribute-concept associations are assessed by combining a target

First, the categories represent concept discrimination: me – not me, or doping-supplements. Then another category pair is used for attribute discrimination – e.g. pleasant -unpleasant.

Finally, the two tasks are combined: semantic targets have to be categorised into one of four categories (two pairs). This combined task is presented twice, the change in the repeat presentation is the reversal of response keys for one category pair (e.g. first RH response is required to 'pleasant' category and LH response to 'unpleasant' category, then on the repeat presentation LH response is required to 'pleasant' and RH response to 'unpleasant'). The IAT assumes that the simultaneous presentation of the two tasks makes strongly associated (compatible) attribute-concept pairs easier (and hence, faster) to classify when their responses are mapped on the same response keys [69]. Given the instruction to respond with maximum speed and accuracy, response times in combined tasks are assumed to depend on how compatible the categories on each side of the computer screen are in peoples' minds [21]. For example, the *me-unpleasant *combination has been shown to produce slower response times than the *me-pleasant *[64]. Further, the difference in response times between the two combined tasks (the initial and the reversed one) were shown to vary substantially across the sample, and were conceptualised as representing the underlying individual differences in the corresponding implicit self-esteem [64]. The response time difference, or the IAT effect [21], essentially constitutes an estimate of the strength of the subject's implicit attitude.

### Aims

The IAT method has been particularly effective in capturing associations with evaluative attributes (e.g. pleasant versus unpleasant) and thus in measuring implicit affect/attitudes. The method also has the potential to deal with more descriptive attribute dimensions (such as trait descriptors) which may result in a meaningful assessment of implicit individual differences [22]. Categories in the semantical decision tasks of the IAT can be constructed to represent forms of performance enhancement that are acceptable (e.g. nutritional supplements) or unacceptable, such as anabolic steroids, growth hormone and other substances or methods prohibited by the World Anti-Doping Agency. Combining these categories with IAT 's standard connotative evaluation dimension enables one to estimate the magnitude of *automatic evaluative preference *(i.e. bias) [59] in favour or against the performance enhancement categories, which can be interpreted, following the IAT tradition [21], as athletes' implicit attitudes towards banned substances and methods.

Supplement use to enhance performance, in general, is widely accepted in the sporting community. Therefore the key question was the attitude towards banned substances in comparison to permissible supplements. Hence, the aims of this study were to i) adapt the IAT protocol to categories of performance enhancement and ii) to examine the construct and concurrent validity of the PE-IAT by complementing the test with two different self-reported attitude measures and demographic data.

## Method

### Participants

One hundred and eleven undergraduate sports and exercise science students participated in the study, which had been approved by the Faculty Research Ethics Committee. In order to maintain complete anonymity, implied consent was used, which was clearly stated in the introduction preceding the web-based procedure as well as on the paper-and-pencil survey complementing the computerised test. The participation was voluntary and students received no compensation or credit. Due to the nature of their course of study, all participants were familiar with performance enhancing methods and nutritional supplements. Of the 111 participants, 78 students completed both the implicit test and the explicit measures. The sample was predominantly male (83.3%), with the mean age of 21.59 ± 5.89. More than half of the participants (61.2%) in the sample were involved in organised competitive sports.

## Materials

### Implicit measure

**Figure 1:**
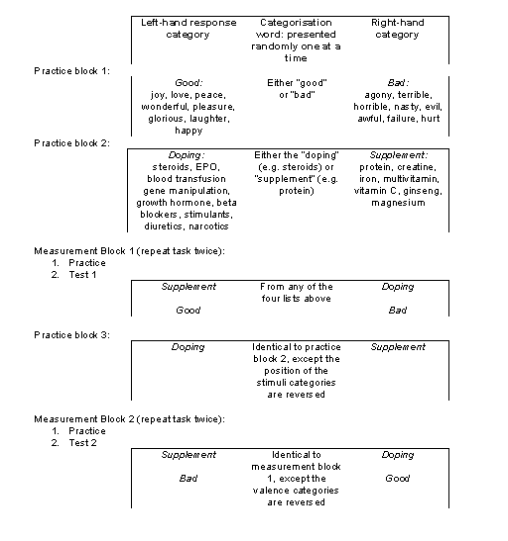
PE-IAT Tasks for the assessment of implicit doping attitudes.

A new category pair (doping vs. nutritional supplements) was added to the conventional 'good vs. bad' IAT stimuli set resulting in the Performance Enhancement IAT (PE-IAT) protocol. The procedure was a web-based dialogue that combined two semantical decision tasks: (a) discriminating between positively and negatively valenced words and (b) discriminating between doping/supplement categories. The good and bad words were selected from the existing IAT lists [21] with a preference for the words that represent clearly valenced emotional states. Following De Houwer [69] and Bluemke and Friese [70], doping-related stimuli were selected to replicate the current official distinction between prohibited methods (doping) and acceptable nutritional supplements [71]. Figure 1 illustrates the PE-IAT procedure and lists all the stimuli used.

Participants completed PE-IAT as shown in Figure 1 in a supervised environment (computer lab) through a web-based protocol delivery. For each participant, six items from each of the four categories were randomly selected. Tests were preceded by short, written descriptions and instructions to sort the words into their respective categories as fast as they can without making a mistake. All words presented in the protocol unambiguously belonged to one of the opposite categories. Incorrectly categorised words were marked with a red cross in the centre of the screen and the task was to be repeated. Sorting was done by pressing either 'e' or 'i' keys on a QWERTY keyboard. Participants were instructed at the beginning of each block to place their fingers on the relevant key and start the test by pressing the space bar when they are ready. Response time was measured in milliseconds for each stimulus. Implicit relative attitude was estimated by the PE-IAT effect, which was calculated as the difference in response latency between the '*good+supplements*' combination and the '*good+doping' *combination [21]. The PE-IAT score is interpreted as an effect in relation to the opposite category: nutritional supplements – doping and vice versa. Whilst the PE-IAT effect can be expressed as an absolute value showing the magnitude of the latency difference, its direction indicates the implicit attitude (more or less preference or aversion) in individual assessment. In this paper, we limit our analyses to groups, where the magnitude of the PE-IAT effect indicates a more or less supportive implicit attitude towards prohibited substances in comparison to nutritional supplements.

First, participants practiced the initial target concept discrimination with doping/nutritional supplements words. Secondly, the attribute discrimination was practiced with good/bad words. Then the two tasks were combined: both pairs of category labels appeared on either left hand (LH) or right hand (RH) side of the screen simultaneously; and target words were randomly selected from either doping/supplement or good/bad word lists and exposed one at a time in the middle of the screen. This combined task was administered twice (Figure 1). Firstly with 'bad' and 'doping' categories on the RH side of the screen, and 'good' and 'supplement' categories on the LH side; and secondly with the attribute category pair reversed on the screen such that the 'doping' category remains on the RH side of the screen but this time next to the 'good' category; while the 'bad' category is shifted to the LH of the screen next to the 'supplement' category. Results from the first paired blocks were practice tests and as such, excluded from the analysis.

### Explicit measures

The PE-IAT was complemented by two self-report measures, the Performance Enhancement Attitude Scale (PEAS) [72] and the 'Five Doping Scenarios Test' (5-DST) modified from Tangen and Breivik [73]. PEAS is a 17-item, six-point Likert-type scale, with statements like 'doping is necessary to be competitive' or 'the risks related to doping are exaggerated' and responses ranging from strongly disagree (1) to strongly agree (6). Previously reported PEAS reliability was above acceptable [61,72], with good internal consistency observed in the current sample (α = .80). The 5-DST utilises 5 competitive scenarios which present varying degrees of certainty about the opponent's doping behaviours and the respondent is asked whether they would resort to doping. The situations range from almost certainty that the opponent does not use doping to almost certainty that he/she does. The five doping scenarios formed a scale with acceptable reliability (*KR-21 *= .68). Additional questions inquired about various aspects of doping and nutritional supplements. Participant were also asked whether they think doping should be legalized – either for top level or all athletes – and whether they think doping is used and is necessary for winning in today's high performance sport.

## Results

**Figure 2:**
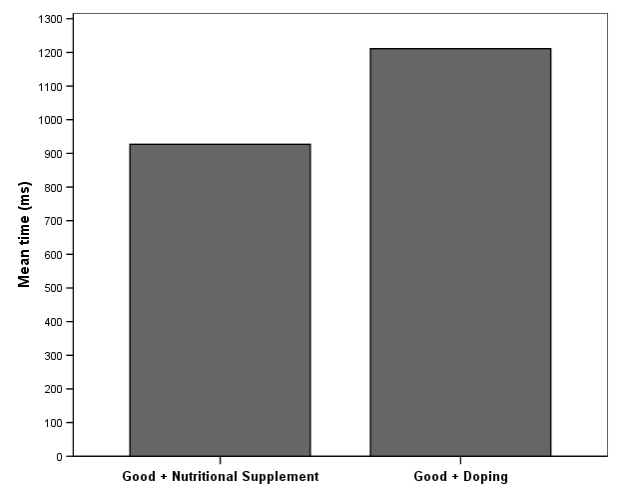
**Observed PE-IAT effects in the sample (n = 96).** PE-IAT effect is the difference in response time between congruent and incongruent pairs = 284.18 ± 244.76.

In accordance with the IAT convention [21,60], response times below 300 and above 3000 were capped at these values and the IAT effect was calculated as a difference between the two tasks [21]. As can be seen in Figure 2, the response time to the combinations of *good+doping *was slower than to the *good+supplement *combination. The average PE-IAT effect was 284.18 *ms*. The difference between *good+doping *and *bad+doping *was statistically significant (*t *= -11.376, *p *< .001), and showed a predictable evaluative bias against doping, i.e. a less favourable automatic evaluation of drugs compared to nutritional supplements. The Guttman split-half coefficient of the PE-IAT was .659. It must be noted that assessments based on response time are inherently more susceptible for error variance; hence the reliability coefficients tend to be lower than those based on explicit measures [74].

**Figure 3:**
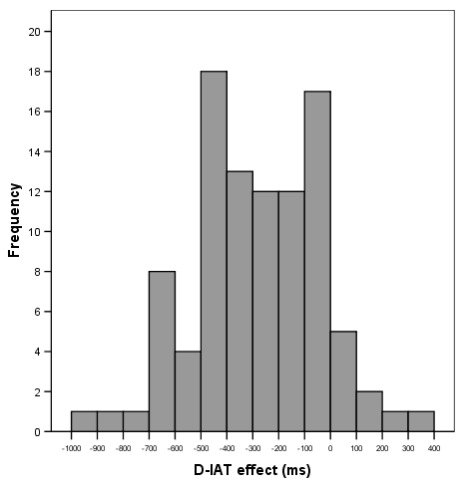
Distribution of the PE-IAT effect.

**Figure 4:**
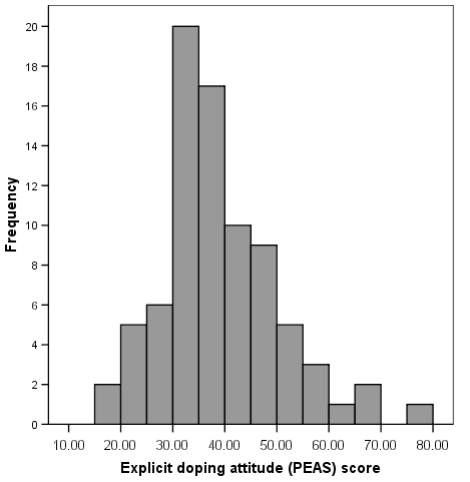
Distribution of the explicit doping attitude (PEAS) scores.

The sample distributions of PE-IAT effect scores (Figure 3) and the explicit attitude (PEAS) score (Figure 4) were near-Gaussian (Kolmogorov-Smirnov *Z *= .578, p = .892 and *Z *= .945, *p *= .333 respectively), indicating that PE-IAT effect is likely to be normally distributed in the population. Normal distribution usually reflects substantive individual differences on the construct – for example, some individuals are more biased against doping than others (Figures 3 and 4). Whilst the PE-IAT test showed a lower preference for doping over supplements, 9 participants (10%) actually presented a stronger association with doping (i.e. latency measures were faster when doping and good were combined than for the opposite task of doping and bad). However, owing to the order in which tasks were presented, individual more lenient IAT effects might have been inflated by the learning effect hence should be interpreted with caution.

A predictable correlation between age and response time in PE-IAT task was observed (*r *= .315, *p *= .013), indicating that older participants were generally slower in responding to the PE-IAT task. PE-IAT effect, however, did not show any association with age (*r *= -.063, *p *= .616) nor differed by gender (*t *= -1.27, *p *= .210). Meaningful differences in PE-IAT effect were observed, between those who report competitive involvement (mean PE-IAT effect of 320.96 ms ± 243.74) and those who do not (mean PE-IAT effect of 263.04 ms ± 226.68). The observed difference was in the predictable direction (competitive athletes are stronger in their implicit disliking of doping) and it is consistent with explanations based on the attitude importance concept [75,76]. However, the effect size is small (Cohen's *d *= .246) and the observed differences were not statistically significant (*t *= -.953, *p *= .344). Significant difference in PE-IAT effect was also found between those who would use doping under certain circumstances (i.e. when their opponent is using drugs) and those who refused to employ such means (Mann-Whitney *U *= 109.05, *p *= .048) with a larger effect (mean PE-IAT effect = 316.45 ± 238.91 ms) observed in the latter group compared to the mean PE-IAT effect of 143.37 ± 187.37 ms of the 'never use' group. It should be noted that the lack of counterbalance in the sequence of tasks may have potentially reduced the IAT effect (*doping+good *task were performed after the *doping+bad*), assuming that the learning effect enhances the processing speed in subsequent tasks, hence time taken on the non-congruent task may be enhanced by the learning effect. Therefore the observed IAT effect is a conservative (lower) estimate of the potential IAT effect and difference found would likely to be larger if tasks were counterbalanced.

On the whole the PE-IAT showed promise as a method with reasonable content validity. An overall preference for nutritional supplements over the prohibited methods was observed despite the fact that: i) 66% declared a belief that doping helps performance (22% claimed not having the knowledge to answer the question) and ii) 61% of the respondents did not believe that nutritional supplements can offer safe alternatives to doping (14% claimed having no knowledge in this matter). The distribution of PE-IAT effect is near-Gaussian, indicating substantive individual differences. These differences are also related to competitive sport involvement: PE-IAT effect is stronger for those reporting competitive sport involvement, which supports PE-IAT's criterion validity.

The explicit attitude measure scores (PEAS) were also normally distributed (Kolmogorov *Z *= .945, *p *= .333; Figure 4). As was expected, the two self-report measures positively correlated (Kendall *τ *= .410, p < .001). Correlations between the explicit and implicit measures were in the expected direction but non-significant (*r *= .181, *p *= .142), which is consistent with the IAT literature [47]. As can be seen in Table 1, the association was slightly stronger among competitive athletes than among their non-competitive student peers Those who were involved in competitive sport were assumed to have greater awareness of doping issues, which is reflected in their explicit attitude scores (although likely to be strongly moderated by strategic responding effect). The possible distorting effect of strategic responding was more pronounced in the correlation between the PE-IAT effect and the hypothetical doping scenarios in which respondents estimated the likelihood they might consider using doping. Here the association was weaker for the competitive athletes than it was for their non-competitive counterparts indicating a predictable dissociation between declared and implicit attitudes as a function of personal relevance.

**Table 1:** Correlation coefficients between implicit and explicit doping attitude (n = 63)

	PE-IAT effect		PEAS	
		
	Competitive	Not competitive	Competitive	Not competitive
PEAS	.159	.130		
	p = .313	p = .546		
	n = 42	n = 24		

5-DST	.145	.224	.332	.558
	p = .263	p = .191	p = .005	p < .001
	n = 42	n = 24	n = 49	n = 28

PE-IAT = Doping Implicit Association Test, PEAS = performance Enhancement Attitude Scale, 5-DST – 5 Doping Scenarios Test. Correlation coefficients are Pearson product moment correlation coefficients (*r) *except for 5-DST, which is Kendall's tau (τ).

Statistically significant difference was found between the explicit attitude (as indicated by the PEAS score) and participation in organized sports competition (*t *= -2.712 *p *= .008). Interestingly, the mean score was higher for those who do not compete (*M *= 43.14 ± 13.15) than for those who participate in organized sport competition (*M *= 36.16 ± 9.45), suggesting again a stronger response bias effect for competitive athletes.

Expressed endorsement to legalizition of doping for top level athletes was associated with the explicit attitude score (Kruskal-Wallis χ^2 ^= 20.10, *p *< .001). Those who endorsed the idea that doping should be allowed scored higher than those who reported the belief that doping by athletes should not be allowed (Table 2). Significant difference in explicit attitude towards doping was also observed between those who would use doping when certain that their opponent is using drugs, and those who declared they would never employ such means (Mann-Whitney *U *= 53.50, *p *< .001). As expected, the mean score was lower for the latter group (Table 2).

**Table 2:** Mean PE-IAT effects of those who favour legalising doping and those who oppose; and those who would use doping

	Level of agreement	PE-IAT effect	PEAS
Legalising doping for TOP athletes	absolutely not	-306.09 ± 229.00	36.98 ± 10.51
	yes with restrictions	-294.53 ± 289.30	48.50 ± 9.81
	yes without restrictions	-14.50*	45.00*

Legalising doping for ALL athletes	absolutely not	-302.36 ± 227.67	35.27 ± 8.49
	yes with restrictions	-270.76 ± 282.67	54.50 ± 12.56
	yes without restrictions	-	45.00 ± 1.41

Hypothetical use of doping (5-DST)	0	-316.45 ± 258.40	35.74 ± 8.72
	1	-	42.00*
	2	-206.60 ± 185.43	55.71 ± 10.81
	3	13.667 ± 61.64	67.50 ± 13.44

PE-IAT = Doping Implicit Association Test, PEAS = Performance Enhancement Attitude Scale, 5-DST – 5 Doping Scenarios Test. Level of agreement for 5-DST: number indicates the number of the five scenarios in which the respondent would consider using doping.

* n = 1

## Discussion

PE-IAT was designed to capture implicit evaluations of doping substances relative to nutritional supplements. This study has produced mixed evidence in support of this design. First, significant differences in response latencies between the two PE-IAT tasks (*doping+good *vs. *doping+bad*) indicated a predictable evaluative bias, thus supporting PE-IAT's design in principle. The fact that the implicit relative attitude towards doping showed no significant correlation with the explicit measures was in keeping with the literature [38]. From the various explanations for weak or non-significant relationship between implicit and explicit measures, the most obvious explanation is the degree of secrecy and sensitivity. Using banned performance enhancement substances is a controversial, socially stigmatized issue. In addition to the motivational process, cognitive determinants may also influence the explicit and implicit relationship [23]. Intuitively, the discrepancy between these two measures increases with the increase of the amount of information to be processed in explicit judgment.

The observed difference in doping/supplement association with valenced categories of good vs. bad; and the weak correlation between explicit and implicit measures do not necessarily indicate that athletes rely exclusively on one or the other when making doping-related decisions. Implicit associations are likely to form a basis for explicit evaluations as long as it is consistent with the processed information [23] or they are more likely to draw upon both in an iterative re-evaluation process [43]. As performance enhancing behaviours are unlikely to be spontaneous, the main implication of our findings is that athletes with a more preferential implicit attitudes to doping (as estimated by the PE-IAT effect in our study) are likely to be more sensitive to doping-relevant cues in situations they encounter and more action-ready when they detect these cues. These athletes may also be faster in detecting these cues and hence biased towards interpreting ambiguous cues as doping-related. Similarly to findings in alcohol dependence studies [37,77] the observed less favourable attitude towards doping may be explained by extrapersonal influence then own values. To verify or falsify this assumption, a new set of data would require using a personalised version of the PE-IAT [59].

Experimentally created extrapersonal associations [77] have shown a reduced IAT effect when the extrapersonal association was incongruent with the participants' own attitudes compared to the group where participants were given attitude-congruent information. However, the extrapersonal influence manipulation did not affect the personalised version of the IAT, which indicated that the personalised IAT is a more robust measure of implicit attitude resistant to extrapersonal influences. Stable individual differences should be taken into account in predicting situated actions. Implicit attitudes, for example, are known to be a better predictor of behaviour for people with high level of intuition in decision making, whereas explicit attitudes are better predictors for deliberative decision makers [78].

Potential contamination might have occurred by associations stored in memory but irrelevant to individual experience or by framing effects, where a stimulus might have evoked a preferential association if stand alone but turned less preferred when it was framed within the good/bad/nutritional supplements/doping context. Those who were involved in organised competitive sport are likely to have a greater awareness of doping issues and of the allowed vs. banned distinction of performance enhancing methods. Hence, PE-IAT stimuli are likely to have been more familiar to them. This confounding effect of stimulus familiarity is yet to be examined for PE-IAT.

Although it was not statistically significant, the observed non-overlapping variance is likely to represent a genuine difference in implicit attitudes between those involved in competition and those who are not, with the former showing smaller PE-IAT effect indicating relatively higher implicit preference towards doping. The strength of relationship between the implicit and explicit association also differed and was less for those who are involved in competitive sport. Doping is probably a more pertinent issue for those driven by competitive motivation. According to the IRM [43], assessment of a situation is a result of a series of evaluative cycles involving both automatic appraisal and attitudes with the aims of: i) reducing discrepancy between explicit and implicit attitudes and ii) minimizing processing demand. The reduced PE-IAT effect among competitive athletes in our sample suggests that these evaluative cycles may have taken place before, resulting in a higher familiarity with prohibited substances and, hence, in higher preference towards them. Ideally, investigations of doping behaviour should consider both explicit and implicit attitudes. Empirical assessment of both explicit and implicit attitudes among athletes and their support personnel is likely to lead to more informed decisions about tailoring education programmes and other anti-doping interventions.

Doping-related decisions are likely to have a solid degree of irrationality. These irrational influences are known to come from two distinct types of sources: those that individuals are unwilling to admit (deliberately hidden agendas), and those they are unable to articulate at all (automatic evaluations, obsessions, etc.). Separating these two types of influence is a worthwhile objective in social science doping research. The Implicit Association Test, its variations and other tools of implicit assessment promise a potential to discover the 'unspoken preference' behind doping choices and actions. The practical application, however, must be approached with great care. The interpretation of data derived via implicit association tests being a 'bias free' assessment is highly debated in the literature [23]. The PE-IAT or similar tests alone may not be not more useful in predicting behavior than verbal declarations if the behaviour is difficult to predict. A combined and complimentary assessment strategy utilizing various psychological tests is likely to be the way forward in understanding the driving forces behind performance enhancement and doping.

## Conclusion

Despite the increased anti-doping effort, the relative adverse analytical findings have continued to increase [79]. A deeper understanding of decision making processes and athletes' dispositions towards performance enhancement may point sport managers, officials and policy makers towards a better-targeted approach or may even point the anti-doping effort towards radically different directions. New methods that allow researchers to obtain a more objective picture of this phenomenon are critically lacking in sport psychology, management and policy development.

This pilot study shows PE-IAT as a promising tool for future research and anti-doping application. The method has uncovered automatic evaluative bias in the predicted direction at group level and connection to competitive sport involvement at individual level. The study has provided some preliminary evidence that the implicit association measure is able to predict behaviour (in self-reported and hypothetical situations) above and beyond the explicit measures.

The findings from this pilot study may be utilised in prevention and intervention method. A unique advantage of this methodology is in its ability to capture and differentiate between un-declared attitudes towards acceptable and banned performance enhancing substances, which may substantially enhance the international anti-doping research efforts. The potential applications of this new research tool may include assessment of doping-related vulnerability levels, including cross-cultural validation, and individual effectiveness of anti-doping interventions. However, the current version of PE-IAT requires validation and further substantial improvement. Enhancing its construct validity and experimenting with variations of its stimulus set and procedures seems a worthwhile objective for future research. Once the identified deficiencies are mitigated, potential applications of the protocol include assessment for social science doping research as well as targeted anti-doping intervention.

Alcohol-related studies have shown that implicit cognitions play an influential role hence should be taken into consideration when designing intervention programs [31]. Implicit attitudes have thought to reflect long term exposure and be particularly resistant to change. However, recent studies have shown that context can be highly influential and under the same conditions, implicitly and explicitly assessed attitudes can change independently of each other [80].

Whilst the implicit association concept as attitude measure has attracted much interest in the past decade, it has also faced with criticism. Using multiple assessments that include a combination of implicit and explicit measures is a highly recommended approach to help the deconvolution of this dissociation between explicit and implicit tests [81]. Researchers investigating socially sensitive issues such as doping and drug use are encouraged to experiment with implicit measures for two distinct reasons. Tackling the problem from a different angle (i.e. using implicit measures instead of solely rely on self-declaration) might prove to be beneficial in both drug and doping research. In addition, drugs and doping use provide excellent testing grounds to examine the theoretical and methodological aspects of implicit attitudes assessment.

Anti-doping intervention and prevention programmes need to show the effective use of public funding. Theoretically sound and empirically validated diagnostic tools are required to help to identify athlete groups vulnerable to doping. The development of the PE-IAT is a first step into this direction. However, the anti-doping strategy must incorporate actions beyond identification. Given the scarce resources, a targeted approach is desirable. Interventions should ideally target both explicit and implicit associations and the effect of interventions should be measured with a combination of both. Despite the best intentions, intervention programmes without an effect on implicit associations may not produce the desirable effect as implicit attitudes may continue to influence doping expectancies in the vulnerable group.

## Competing interests

The authors declare that they have no competing interests.

## Authors' contributions

EA conceived the study, developed the PE-IAT protocol and helped to draft the manuscript. AP contributed to the development of the PE-IAT protocol, collected data, performed the statistical analyses and drafted the paper. TN developed the web-based PE-IAT test site and managed the database. All authors read and approved the manuscript.
